# Supplementary material for: Functional diversity among sensory neurons from efficient coding principles
Source: PLoS Comput Biol. 2019 Nov 14;15(11):e1007476. doi: 10.1371/journal.pcbi.1007476 (PMC6890262; doi:10.1371/journal.pcbi.1007476)
Supplement: S1 Text — The mutual information for different noise models. (PDF) [file pcbi.1007476.s003.pdf]

S1 Text: Mutual information for a system with two cells

We demonstrate that the equal coding theorem, where any number of ON and OFF cells codes the same information, does not follow trivially from changing ON cells into OFF cells by exchanging a spike for no spike. To illustrate this in the most intuitive way, we compare a system with one ON and one OFF cell with optimally adjusted thresholds θ_1 and θ_2 respectively, and two ON cells also with optimally adjusted thresholds, θ_1 and θ_2. As stated in the main text, we assumed that ON (OFF) neurons fire Poisson spikes with an average mean count $\nu_{\max}$ whenever the stimulus intensity is above (below) their threshold θ_i, and zero otherwise, i.e. $\nu_i(s) = \nu_{\max}\Theta(s-\theta_i)$ for ON neurons and $\nu_i(s) = \nu_{\max}\Theta(\theta_i - s)$ for OFF neurons (Fig. 1B of the main text), where Θ is the Heaviside function. For such response functions with two firing rate levels, 0 and $\nu_{\max}$, we can lump together all states with nonzero spike counts into a single state, which we denote as 1. Correspondingly, we denote the state with zero spikes as 0. We introduce the parameter $q = e^{-\nu_{\max}T}$, which ranges from $q = 0$ in the noiseless limit of high firing rate, and $q = 1$ in the high noise limit of low firing rate. Then,

$$\begin{aligned} p(0|\nu = 0) &= 1, \quad & p(1|\nu = 0) &= 0, \\ p(0|\nu = \nu_{\max}) &= q, \quad & p(1|\nu = \nu_{\max}) &= 1 - q. \end{aligned} \tag{S1}$$

Let (n_1, n_2) denote the output vector, with (ν_1, ν_2) being related to the input since the firing rate of each cell is determined by how the output distribution $p(n_1, n_2)$ is partitioned by the thresholds. For the ON-OFF system, this partitioning has the form:

$$\begin{aligned} (0,0) &\to \theta_2 - \theta_1, \\ (0,\nu_{\max}) &\to 1 - \theta_2, \\ (\nu_{\max}, 0) &\to \theta_1, \end{aligned} \tag{S2}$$

resulting in the conditional probability matrix $p(n_1, n_2|s)$ given in S1 Table.

Table S1. Conditional probability matrix $p(n_1, n_2|s)$ for a mixed ON-OFF system.

Now, we convert one OFF cell into an ON cell. Again, the output distribution $p(n_1, n_2)$ is partitioned by the thresholds, which for the ON-ON system has the form:

$$\begin{aligned} (0,0) &\to \theta_1, \\ (\nu_{\max}, 0) &\to \theta_2 - \theta_1, \\ (\nu_{\max}, \nu_{\max}) &\to 1 - \theta_2, \end{aligned} \tag{S3}$$

resulting in the conditional probability matrix $p(n_1, n_2|s)$ given in S2 Table. It is clear that changing one

Table S2. Conditional probability matrix $p(n_1, n_2|s)$ for a homogeneous ON-ON system.

OFF into an ON cell does not simply exchange the rows of the conditional distribution matrix, but alters them. In particular, while the first and second rows are exchanged between the ON-ON and ON-OFF systems, the third row is very different, regardless of threshold settings. Therefore, we conclude that there is no simple symmetry argument for why the information between the ON-OFF and ON-ON systems should be identical.

The identity of mutual information does not depend on the noise model

We consider two different noise models to demonstrate that the mutual information is very similar in a system of two cells, independent of whether the population is mixed (one ON and one OFF cell), or homogeneous (two ON cells).

Figure S1. A framework with binary neurons that have two firing rate levels, r if the stimulus is smaller (bigger) than a threshold, and R if the stimulus is bigger (smaller) than a threshold for ON (OFF) cells. We compare two systems, left: one ON (red) and one OFF (blue) cells, and right, two ON cells, where the information is maximized by optimizing the cells thresholds. Information results in Table S3.

Table S3. Mutual information for the systems comprised of binary cells, one ON and one OFF cells, vs. two ON cells (see S1 Figure) where the spike counts r and R have been varied, assuming Poisson noise on the spike count. In all cases, the information is nearly identical.

Figure S2. Two sigmoidal nonlinearities for an ON cell (red) and an OFF cell (blue), describing the firing rate as a function of stimulus with the maximum expected spike count R, the gain β, and the threshold θ. The shaded curve denotes the Laplace stimulus probability distribution. Information results in S4 Table.

First, we continue as before with a Poisson noise model, but where we assume that the neural response of an ON (OFF) cell to a stimulus below (above) a certain threshold is nonzero (S1 Figure). The expected spike counts are determined by the firing rate levels whenever the stimulus is smaller (r) or bigger than a threshold (R). Because of this assumption, we can no longer make the simplification from Eq. S1, (also Eq. 12 in the main text) about the way noise enters the system and which allows us to do analytical calculations, but need to explicitly sum over all possible spike counts (up to some cut-off) given r and R, numerically. Therefore, we compared systems of two neurons, one ON and one OFF, vs. ON-ON in which the levels of firing in the high and low state were the same, but where we optimized the thresholds for a Laplace stimulus distribution. We obtained the results in S3 Table for different values of r and R, which confirm our initial conclusion about equality of ON-OFF and ON-ON coding.

Second, we considered sigmoidal nonlinearities fitted from recorded salamander retinal ganglion cells [1, 2]. Since real ganglion cells do not conform exactly to Poisson statistics [3, 4], we considered an output noise distribution that matches the observed sub-Poisson noise in spike counts of salamander retinal ganglion cells, as measured experimentally [1]. The form of the nonlinearity was

$$g_{\mathrm{ON}}(s) = R\frac{1}{1+e^{-\beta(s-\theta_{\mathrm{ON}})}} \quad \text{and} \quad g_{\mathrm{OFF}}(s) = R\frac{1}{1+e^{\beta(s-\theta_{\mathrm{OFF}})}} \tag{S4}$$

for ON and OFF cells, respectively.

These sigmoids have gain β and a maximal spike count of R (S2 Figure). In this model, we assumed that the output noise distribution was sub-Poisson, fitted directly form data [1]. In particular, for a given mean spike count c, the measured spike count distribution $p(k|c)$ has width that stayed constant with c after an initial Poisson-like growth ([1] (see their Supp. Fig. 3). The distribution is well described by the heuristic formula

$$p(k|c) \propto \exp\left[-\frac{(k-k_0(c))^2}{2\sigma^2}\right], \quad k = 0, 1, 2, \dots \tag{S5}$$

where

$$k_0(c) = a\log(-1+e^{c/a}), \tag{S6}$$

with $a = 0.5$ nd $\sigma = 0.75$ [1]. Using this noise model, we computed the mutual information numerically, and had to sum over the spike counts for each cell (up to some cutoff). The fitted sigmoids from the data from salamander ganglion cells had gains of $\beta = 5.8$ (expressed in inverse of the standard deviation of the stimulus), and $\beta = 2.3$ from the data from macaque ganglion cells. The maximal firing rates were also extracted from the data to be: median peak firing rate for salamander ganglion cells of 48 Hz, which with a coding window of 50 ms translates to $R = 2.4$, and median peak firing rate for macaque ganglion cells of 220 Hz, which with a coding window of 10 ms translates to $R = 2.2$. Based on these fits, we computed the mutual information as before for a system with two cells, one ON and one OFF, and two ON cells (S4 Table), demonstrating that the mutual information was identical.

Table S4. Mutual information for the systems comprised of neurons modeled as sigmoidal nonlinearities with sub-Poisson noise measured empirically in the retina, for two systems, one ON and one OFF cells, vs. two ON cells (see S2 Figure). The maximal firing rates and gains were extracted from the data. In all cases, the information is identical.

References

1. Pitkow X, Meister M. Decorrelation and efficient coding by retinal ganglion cells. Nat Neurosci. 2012;15:628–635.

2. Gjorgjieva J, Sompolinsky H, Meister M. Benefits of pathway splitting in sensory coding. J Neurosci. 2014;34:12127–12144.

3. Berry MJ, Meister M. Refractoriness and neural precision. J Neurosci. 1998;18:2200–2211.

4. Uzzell VJ, Chichilnisky EJ. Precision of spike trains in primate retinal ganglion cells. J Neurophysiol. 2004;92:780–789.
