# Supplementary material for: Functional diversity among sensory neurons from efficient coding principles
Source: PLoS Comput Biol. 2019 Nov 14;15(11):e1007476. doi: 10.1371/journal.pcbi.1007476 (PMC6890262; doi:10.1371/journal.pcbi.1007476)
Supplement: S1 Table — Conditional probability matrix p(k1, k2|s) for a mixed ON-OFF system. (PDF) [file pcbi.1007476.s004.pdf]

Table S1. Conditional probability matrix $p(n_1, n_2|s)$ for a mixed ON-OFF system.

(n_1, n_2) / stimulus regime	$(0,0)$	$(0,1)$	$(1,0)$	$(1,1)$
θ_1	q	0	$(1-q)$	0
$\theta_2 - \theta_1$	1	0	0	0
$1 - \theta_2$	q	$(1-q)$	0	0
