# Supplementary material for: Functional diversity among sensory neurons from efficient coding principles
Source: PLoS Comput Biol. 2019 Nov 14;15(11):e1007476. doi: 10.1371/journal.pcbi.1007476 (PMC6890262; doi:10.1371/journal.pcbi.1007476)
Supplement: S3 Table — Mutual information for a two-cell system with spontaneous firing rate and Poisson noise. (PDF) [file pcbi.1007476.s006.pdf]

Table S3. Mutual information for the systems comprised of binary cells, one ON and one OFF cells, vs. two ON cells (see S1 Figure) where the spike counts r and R have been varied, assuming Poisson noise on the spike count. In all cases, the information is nearly identical.

Information (ON-OFF vs. 2ON) in bits	$R = 1$	$R = 10$
$r = 0.2R$	0.244 vs. 0.242	0.959 vs. 0.958
$r = 0.5R$	0.0795 vs. 0.0792	0.529 vs. 0.527
