# Supplementary material for: Functional diversity among sensory neurons from efficient coding principles
Source: PLoS Comput Biol. 2019 Nov 14;15(11):e1007476. doi: 10.1371/journal.pcbi.1007476 (PMC6890262; doi:10.1371/journal.pcbi.1007476)
Supplement: S4 Table — Mutual information for a two-cell system with empirically measured sub-Poisson noise from salamander retinal ganglion cells. (PDF) [file pcbi.1007476.s007.pdf]

Table S4. Mutual information for the systems comprised of neurons modeled as sigmoidal nonlinearities with sub-Poisson noise measured empirically in the retina, for two systems, one ON and one OFF cells, vs. two ON cells (see S2 Figure). The maximal firing rates and gains were extracted from the data. In all cases, the information is identical.

Information (ON-OFF vs. 2ON) in bits	salamander	macaque
	1.24 vs. 1.24	0.865 vs. 0.865
